# Atomic Partitioning of the MPn (*n* = 2, 3, 4) Dynamic Electron Correlation Energy by the Interacting Quantum Atoms Method: A Fast and Accurate Electrostatic Potential Integral Approach

**DOI:** 10.1002/jcc.26037

**Published:** 2019-08-02

**Authors:** Mark A. Vincent, Arnaldo F. Silva, Paul L. A. Popelier

**Affiliations:** ^1^ Manchester Institute of Biotechnology The University of Manchester Manchester M1 7DN UK; ^2^ School of Chemistry The University of Manchester Manchester M13 9PL UK

**Keywords:** FFLUX, QTAIM, QCT, electron correlation, Moller Plesset

## Abstract

Recently, the quantum topological energy partitioning method called interacting quantum atoms (IQA) has been extended to MPn (*n* = 2, 3, 4) wave functions. This enables the extraction of chemical insight related to dynamic electron correlation. The large computational expense of the IQA‐MPn approach is compensated by the advantages that IQA offers compared to older nontopological energy decomposition schemes. This expense is problematic in the construction of a machine learning training set to create kriging models for topological atoms. However, the algorithm presented here markedly accelerates the calculation of atomically partitioned electron correlation energies. Then again, the algorithm cannot calculate pairwise interatomic energies because it applies analytical integrals over *whole* space (rather than over atomic volumes). However, these pairwise energies are not needed in the quantum topological force field FFLUX, which only uses the energy of an atom interacting with all remaining atoms of the system that it is part of. Thus, it is now feasible to generate accurate and sizeable training sets at MPn level of theory. © 2019 The Authors. *Journal of Computational Chemistry* published by Wiley Periodicals, Inc.

## Graphical abstract

As pointed out by Shakespeare, the QTAIM man has a hard choice to make: using the 3D ESP approach enhances the algorithm speed by a factor of six and offers increased accuracy, but information is lost on how a given atom A interacts with another atom B. Still, the developing force field FFLUX needs only the overall (summed) interaction between atom A and all other atoms. Hence, the 3D ESP algorithm is an important advance for FFLUX.

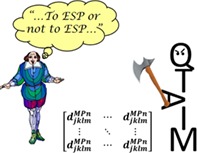

## Introduction

Atomistic force fields continue to be developed^1^, ^2^, ^3^, ^4^, ^5^, ^6^, ^7^, ^8^, ^9^, ^10^ given their growing use and impact in biological and materials science. Extensive comparisons^11^, ^12^, ^13^, ^14^ between, traditional force fields can offer guidance in this development, as well as comparisons^15^, ^16^ between machine‐learnt potentials. Within this grand scheme of force field development, the modeling of dispersion^17^ energy has received special attention over the last decade and more.

Dispersion is normally defined within the context^18^ of long‐range Rayleigh‐Schrödinger perturbation theory, which is typically used in the description of two or more interacting molecules. However, it is possible to circumvent perturbation theory and focus directly on the electron correlation, from a single supermolecular system. This is the route we follow here. We calculate dynamical correlation energy extracted from the two‐particle density‐matrix (2PDM) of this single supermolecular (quantum) system. This energy encompasses dispersion effects. We thus apply an alternative method to obtain a dispersion‐like interaction energy between molecules.

A real‐space partitioning, such as the quantum topological one,^19^ of the energy associated with electron correlation allows dispersion to be quantified within a single atom. As a result, intra‐molecular dispersion is also defined and quantifiable, which is not the case within a perturbation approach. The capacity to look inside an atom and assess the extra stability it experiences due to electron correlation yields deeper insight into the phenomenon of dispersion, beyond that offered by the traditional Lennard‐Jones intermolecular dispersion. In this work, we adopt the (topological) real‐space method of interacting quantum atoms (IQAs)^20^ to provide dispersion energies, both within a given atom and between *any pair of* atoms.

Already in 2005, quantum topological correlation energies were calculated^20^ for a few very small molecules at full CI level (for H_2_ and He_2_) and for complete active‐space multiconfigurational wave functions. Much later, the first IQA‐partitioned electron correlation energies were computed in the context of coupled cluster theory,^21^ coupled‐cluster Lagrangian densities^22^ and CCSD(T) wave functions.^23^ Very recently, we carried out IQA‐MPn for the first time,^24^, ^25^ applied^26^ it to the machine learning method Gaussian Process Regression (also known as kriging), quantified^25^ electron correlation of the chemical bond (showing that ionicity and covalency are not each other's opposite), demonstrated the transferability of topologically partitioned electron correlation energies in water clusters,^27^ studied^28^ the effects of higher orders of perturbation theory on the correlation energy of atoms and bonds in molecules, and finally calculated^29^ dynamic electron correlation in a wider variety of systems including glycine…water (hydration), the ethene dimer (π–π interactions), benzene (aromaticity), cyclobutadiene (antiaromaticity), and NH_3_BH_3_ (dative bond).

The work just described focuses on chemical insight and hence the most detailed information possible, that is, obtained at full atomic resolution. Indeed, IQA‐MPn then gives access to all interatomic energies (denoted A,B) and all intra‐atomic energies (denoted A,A) where A and B are (topological) atoms. In other words, the interatomic energies are known between any two atoms *A* and *B* wherever they are in the system; the atoms do not need to be bonded. Second, it should be emphasized that this information is separated from the intra‐atomic one. This high‐resolution contrasts with the coarser atomic resolution that is the subject of the current article. Here, we only discern the *total* interaction of a given atom with all other atoms in the system (denoted AA’), including its self‐interaction (denoted AA). This restriction is the price paid for the faster algorithm presented here, and the reason for this price will become clear in the next section. However, such overall interaction between a given atom and its environment is sufficient for the development of our quantum topological based force field, called FFLUX.^30^, ^31^, ^32^, ^33^, ^34^ This force field uses kriging to deliver fully polarizable multipolar electrostatics alongside intra‐ and interatomic nonelectrostatic energies and charge transfer as a function of flexible molecular geometries. After all, FFLUX only needs to know the extent by which a given atom interacts with all other atoms. This atomic information suffices to calculate the force^35^ on the nucleus of the atom in question, which then allows the propagating of the system in time, leading to optimized geometries or structural and thermodynamic properties in the case of a molecular dynamics simulation. Indeed, a force field always sums all the individual pairwise interactions that a given atom is involved in. It is only this sum that is needed. Knowing the individual terms would only be useful if detailed chemical insight were being kept track of, which can be done^34^ in principle but this is computationally expensive.

In summary, we present a fast computational procedure in order to equip the topological force field FFLUX with dynamic electron correlation and thus any dispersion effects. This procedure involves an analytical integration over whole space and thereby reduces the expensive six‐dimensional (6D) integration to a three‐dimensional (3D) integration.

## Background and Method

### Derivation

We have presented our original approach in detail elsewhere,^24^ which we briefly summarize here. The key equation is eq. (1),

(1) $$V_{\mathrm{ee},\text{corr}}^{\mathrm{AB}}=\sum_{j=1}^{N_{\text{basis}}}\sum_{k=1}^{j}K_{\mathrm{jk}}\sum_{l=1}^{N_{\text{basis}}}\sum_{m=1}^{l}K_{\mathrm{lm}}d_{\text{jklm}}^{\text{corr}}\int_{\Omega_{A}}dr_{1}G_{\mathrm{jk}}\left(r_{1}-R_{\mathrm{jk}}\right)\int_{\Omega_{B}}dr_{2}\frac{1}{r_{12}}G_{\mathrm{lm}}\left(r_{2}-R_{\mathrm{lm}}\right)$$

where *V* is the (electron) correlation energy between any two atoms *A* and *B* (note that the intra‐atomic correlation energy, that is, when *A = B*, is also covered), *d* is the correlated part of the 2PDM, the *G* functions are Gaussians arising from products of Gaussian primitives originally centred at nuclei, *K* refers to the product pre‐factor (and should of course not be confused with summation index *k*), and there are *N*_basis_ Gaussian primitives. There are two consecutive 3D integrations, each over the volume of a topological atom Ω, introducing its own integration variable (**r**_1_ or **r**_2_). Traditionally, the resulting 6D integration is carried out completely numerically, after installing a quadrature grid (a grid, in short) over each of the respective atomic 3D volumes.

Now we show how an analytical integration over whole space can be introduced. The starting point of the derivation below is the calculation of Vee,corrAA', which is obtained as the sum of the contributions from all atoms *B*. Substituting eq. (1) into this sum leads to the derivation shown in eq. (2),(2)
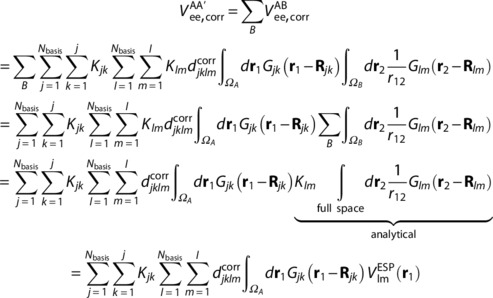
where the summation index B is allowed to be equal to A (and indeed must be in one term of the summation), and where K_lm_ is absorbed in $V_{\mathrm{lm}}^{\mathrm{ESP}}$. We stress that the summation over B is *not* restricted in the familiar sense of A < B or A ≠ B, for example. Indeed, the analytical integration demands integration over *whole* space, and thus B must run over *all* atoms, without restriction. Second, we note that, although not shown in eq. (2), the actual implementation of eq. (2) halves the value of $V_{\mathrm{ee},\text{corr}}^{\mathrm{AB}}$ for the case of A ≠ B only. The introduction of this factor of ½ avoids double counting, that is, if A has interacted B then B has already interacted with A.

The transformation from the second to the third line in the above derivation is justified by the linearity of integration (i.e., the sum of an integral is the integral of the sum, in short) and the distributivity of summation versus multiplication.

It is clear in the third line that the partition over atom *B* disappears and thus the integral is carried out over whole space. Hence, the integral can be calculated analytically, using ideas reminiscent of the calculation of the electrostatic potential (ESP). This integral technology has matured some time ago culminating in the elaborate PRISM algorithm,^36^ which has been implemented in the program GAUSSIAN09^37^ (or G09, in short) used in this work. Note that, although we use the label ESP, we actually do *not* calculate the electrostatic potential itself. The latter involves the electron density (in the integrand) rather than a Gaussian, as is the case in eq. (2). Indeed, the electrostatic potential is a molecular property, but here we face an integral involving only atomic orbitals. Note also that the penultimate expression in eq. (2) cannot be re‐written such that the actual ESP appears. In summary, the net advantage is that a numerical grid, one for each atom, has disappeared, and with it an expensive integral.

There are two additional comments to be made here. First, the main idea of replacing a collection of atomic integrations, each over a numerical grid, by a single analytical integration over whole space, is also at the basis of the so‐called AA' algorithm in the program AIMAll,^38^ which uses it to calculate Coulomb and exchange energies very accurately and quickly. Second, although we explain the algorithm in connection with the program GAUSSIAN09, the algorithm is general and could be interfaced with any quantum chemical software offering efficient computation of ESP, or even with a public library that contains all possible Gaussian primitive integrals.

The purpose of this article is to quantify the improvement over the original 6D algorithm of the new ESP‐based algorithm (discussed in detail below). A simple measure that helps in achieving this is the so‐called *recovery error*, which is defined as the difference between the system's correlation energy directly obtained from G09 and that obtained (i.e., recovered) from the sum of atomic contributions Vee,corrAA', or

(3) ΔEee,corr=Vee,corrG09−∑AVee,corrAA'

We will report this recovery error for a wide variety of systems, alongside with estimates of CPU timings. It should be pointed out that the summation over A is not restricted at all. This lack of restriction does not cause double counting because this issue has already been dealt with in connection with eq. (2) and its discussion. To be more precise, the unrestricted summation over A generates AB and BA but the halving of these contributions (see eq. (2)) compensates for this double counting.

### Implementation of the 3D ESP algorithm

In order to evaluate eq. (2), we have modified a link of G09 (called L1111) to generate the correlated part of the 2PDM (denoted *d*). In addition, the standard wave function file (“wfn”) is outputted from G09. These two pieces of information are then passed to our in‐house code called MORFI, which generates a grid for the molecule under study, atom by atom. This grid (over Ω_A_) is then passed back to G09 to generate the ESP integrals^39^ at each grid point **r**_1_ (see eq. (2)). The nuclear part of an ESP integral is not needed. Because only the electronic part is required we have further modified G09 to obtain these values. The latter are then used as input for another MORFI run, where the atomic energies are calculated from *d* via eq. (2).

**Figure 1:**
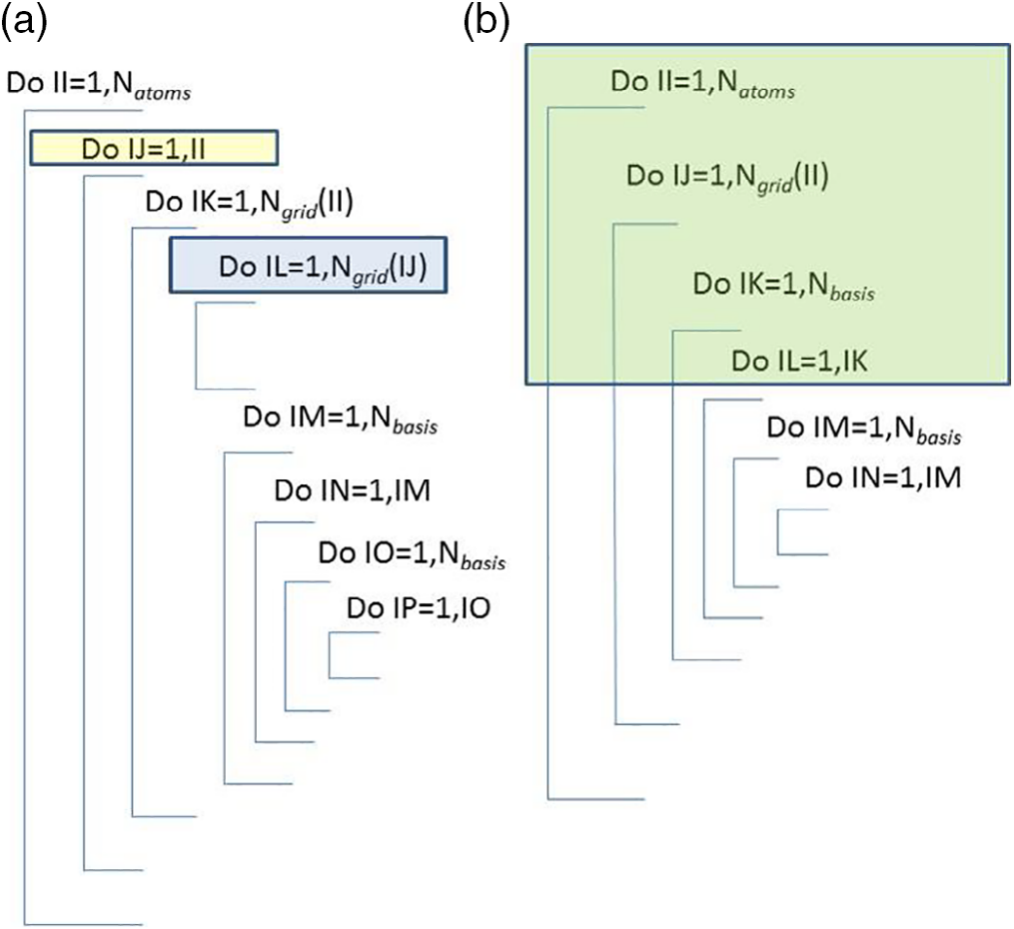
A schematic representation of the DO‐loops involved in the running of the program MORFI by the (a) original 6D algorithm and the (b) new 3D ESP algorithm. The two DO‐loops missing in the latter (but present in the former), are indicated in (a) as yellow and blue. In addition, the DO‐loops indicated in green in (b) actually correspond to the total number of ESP integrals. Hence the green part can be thought of as a single repeated action consisting of reading and processing the ESP integrals. [Color figure can be viewed at http://wileyonlinelibrary.com]

Figure 1 compares the implementation of the original 6D algorithm (left, $V_{\mathrm{ee},\text{corr}}^{\mathrm{AB}}$) with that of the new *3D ESP algorithm* (right, Vee,corrAA'). Both implementations consist of a nested DO‐loop structure, introducing three numbers: *N*_atoms_, *N*_grid_, and *N*_basis_. These counters respectively refer to the number of atoms, the number of (quadrature) grid points of each atom and the number of Gaussian primitive basis functions. The original 6D algorithm is computationally expensive because it involves a twofold nested DO‐loop over the number of atoms covering all atom‐atom pairs (including AA self‐interaction) (*N*_atoms_(*N*_atoms_ + 1)/2 or approximately $N_{\text{atoms}}^{2}$), one DO‐loop over the grid points installed on each atom's volume (*N*_grid_) (hence two nested DO‐loops in total), and four DO‐loops over the number of basis functions ($\left[\frac{N_{\text{basis}}\left(N_{\text{basis}}+1\right)}{2}\times \frac{N_{\text{basis}}\left(N_{\text{basis}}+1\right)}{2}\right]\approx \frac{1}{4}N_{\text{basis}}^{4}$). As a result, the number of executions of the innermost DO‐loop (over index IP in Fig. 1) scales roughly as $N_{\text{atoms}}^{2}N_{\text{grid}}N_{\text{basis}}^{4}$. In addition, there is another DO‐loop (over index IL in Fig. 1) over the grid that does not include the $N_{\text{basis}}^{4}$ DO‐loops. This large number of DO‐loops has confined the application of $V_{\mathrm{ee},\text{corr}}^{\mathrm{AB}}$ to small systems only, with some of our larger systems studied being the water pentamer,^27^ glycine^28^ and glycine…water.^29^

In summing over the atoms, one obtains a *total* energy of an atom in the molecule rather than a self‐energy plus all the pairwise energies of that atom with all the other atoms in the molecule. Thus, the decrease in computational effort demands as a price the loss of detailed energy contributions. Naively, one would expect a massive speed‐up, by a factor of several thousands, due to the elimination of several grid per atom (i.e., *N*_atom_*N*_grid_) and *N*_grid_ is of the order of a thousand. However, this is not quite the case. Indeed, there are actually approximately $N_{\text{atom}}N_{\text{grid}}N_{\text{basis}}^{2}/2$ ESP integrals to be calculated, which is a significant number. Then again, we have replaced one numerical 3D volume integration by, in theory, an analytic one, which means we expect increased numerical accuracy.

We have employed the uncontracted 6‐31++G(2d,2p) basis set with the MP4SDQ (including core orbitals) method to study a series of molecules and complexes. The basis sets are in their uncontracted form because MORFI works in primitive functions and therefore a contracted basis set offers no advantage in the most time‐consuming stage of the calculation. Indeed, it takes longer to generate the MP4SDQ wave function using the uncontracted basis and to optimize the geometry but generally this extra effort lengthens the overall calculation time by a relatively small amount. Although we employ the MP4SDQ 2PDM in this study, any correlation method's 2PDM can actually be employed. We have used the MP2 and MP3 methods to generate the respective 2PDM in our previous studies.^25^, ^28^ In these cases, the density matrices are the same size as the MP4SDQ 2PDM. Only the matrix elements differ, meaning that the calculation time in MORFI is the same irrespective of the wave function. However, MP2 can sometimes have more zero matrix elements than MP3 and MP4SDQ.

Our aim is twofold: (1) to understand how the size of the grid affects the recovery error when the 3D ESP approach is employed and (2) for a fixed recovery error, to find out how much faster the 3D ESP approach is than the original 6D approach. For this purpose, we conducted two types of calculations, one for each aim, respectively, discussed in sections Grid size to achieve a given recovery error and Algorithm speed up (from 6D to 3D ESP) for a fixed recovery error.

## Results and Discussion

### Grid size to achieve a given recovery error

We first consider the size of grid necessary *to achieve a given recovery error* in the correlation energy of the whole system. Note that the FFLUX force field aims for the recovery error be of the order of 1 kJmol^−1^ prior to training. Table 1 gives some grid sizes and recovery errors for the water dimer, F_2_ and the neon dimer, which have all been geometry‐optimized.

**Table 1:** The recovery errors (calculated by MORFI) of the MP4SDQ energy of the water dimer, F_2_ and the neon dimer (Units: kJmol^−1^).

Grid n_ang_‐n_rad_ a	No grid points^b^	MP4SDQ Correlation energy of G09	6D recovery error (MORFI)	3D ESP recovery error (MORFI)
Water dimer				
1‐10	120	−1508.3	+772.8	+2.5
3‐10	520	−1508.3	−31.6	−0.5
5‐10	1000	−1508.3	−68.0	+0.2
10‐10	3400	−1508.3	−39.0	+0.1
5‐20	2000	−1508.3	+37.8	−0.1
10‐20	6800	−1508.3	−17.4	−0.1
F_2_				
1‐10	120	−1992.4	+274.2	+15.4
3‐10	520	−1992.4	−57.2	−0.05
5‐10	1000	−1992.4	−59.1	+0.5
10‐10	3400	−1992.4	−11.8	+0.2
Neon dimer				
1‐10	120	−1388.7	−586.1	+20.3
3‐10	520	−1388.7	−233.7	+20.2
10‐10	3400	−1388.7	43.9	+20.2
15‐10	7000	−1388.7	102.0	+20.2
10‐20	6800	−1388.7	−49.2	+0.4
15‐20	14,000	−1388.7	−22.5	+0.4
10‐30	10,200	−1388.7	−40.1	−0.0
15‐30	21,000	−1388.7	−24.2	−0.0
20‐40	47,200	−1388.7	−14.6	+0.0

a In the notation *n*_ang_‐*n*_rad_ the first number is the Lebedev angular part of the grid while the second number is the radial part of the grid, given by Gauss‐Legendre quadrature. The number *n*_ang_ refers to a grid shorthand adopting the values 1, 3, 5, 10, 15, or 20, which respectively designate angular grids of 6, 26, 50, 170, 350, and 590 points. The number *n*_rad_, on the other hand, directly designates the number of radial points in the Gauss‐Legendre grid.

b The total number of grid points is the number of Gauss‐Legendre radial points multiplied by the number of Lebedev angular points, multiplied by two (one grid inside the β‐sphere and one outside the β‐sphere).

Table 1 contrasts the recovery errors obtained with the new 3D ESP integration with those of the original 6D method, for a variety of grids. It is clear that the errors are dramatically reduced in going from 6D to 3D, by one to two orders of magnitude. Table 1 also shows that, for the neon dimer, the recovery error of the 3D ESP method follows the radial part of the grid and not the angular part (so *n*_rad_ in *n*_ang_‐*n*_rad_). Indeed, the recovery errors are nearly constant within each of the *n*_ang_‐10, *n*_ang_‐20, and *n*_ang_‐30 sets of grids. However, this observation is not true for the water dimer or for F_2_. Nevertheless, the error generally becomes smaller for all three systems, for both 6D and 3D ESP integration, with increasing angular grid. For the neon dimer, the 10‐20 grid, with the 3D ESP approach, yields energies with an error of 0.4 kJmol^−1^, while for the water dimer and F_2_ errors of −0.5 and − 0.05 are, respectively, observed for the 3‐10 grid. These are acceptable errors in the context of the FFLUX force field. However, they do imply that the electronic structure of different molecules call for different grids in order to obtain a similar absolute accuracy in the recovery error with the 3D ESP method. For example, 3‐10 for (H_2_O)_2_ as opposed to 10‐20 for Ne_2_.

In Table 2, we present the results for a variety of molecules studied with the 10‐20 grid only. This grid was selected because for the neon dimer it was the smallest grid that already gave an acceptable recovery error by the 3D ESP algorithm (Table 1, 0.4 kJmol^−1^). In general, a comparison between Tables 1 and 2 shows that the large recovery errors appear for the non‐ESP (6D) approach and for small grids. Furthermore, our modest 10‐20 grid yields reasonable accuracy in most cases (via the ESP approach). The large errors from the non‐ESP (6D) approach stem from two numerical integrations, while in the ESP (3D) approach, one of these is replaced by analytic integration [eqs. 1, 2]. By consistently using the 10‐20 grid in Table 2, which is larger than some of the grids used in Table 1, smaller recovery errors are generally listed in Table 2. Thus, the 3D ESP approach significantly reduces the recovery error compared to the original 6D method for all systems, except for BH_3_ and BeH_3_^−^. This reduction is typically at least one order of magnitude but often two orders. However, for BH_3_, BeH_3_^−^, there is barely a reduction. In absolute terms, four systems show an error of more than 1 kJmol^−1^ (BH_3_, BeH_3_^−^, AlH_3_, and MgH_3_^−^), and five systems (Ne_2_, CO, SiH_4_, HCl, and FHF^−^) show medium sized errors (0.4–0.7 kJmol^−1^).

**Table 2:** The recovery errors (kJmol^−1^) for the 6D and 3D ESP integration for a variety of systems. All results have been calculated with the 10‐20 grid (170 angular Lebedev points and 20 radial Gauss‐Legendre points, or 6800 points in total, for the volume inside and outside the β‐sphere).

System (Molecule or complex)	MP4SDQ energy of G09	6D recovery error (MORFI)	3D ESP recovery error (MORFI)
LiH	−177.4	−3.5	−0.1
BeH_3_^−^	−383.3	+2.9	+2.4
BH_3_	−423.5	+2.1	+1.1
CH_4_	−635.3	−1.5	−0.0
NH_3_	−707.5	−5.9	−0.0
H_2_O	−752.0	−9.5	−0.0
HF	−751.9	−13.1	+0.1
N_2_	−1130.4	−14.0	−0.0
O_2_	−1266.1	−14.2	−0.0
F_2_	−1456.6	−23.8	−0.1
Ne_2_	−1388.7	−49.2	+0.4
NO	−1186.0	−16.1	−0.0
CO	−1087.7	−17.3	−0.4
OF	−1302.5	−19.7	−0.1
NaH	−575.0	−15.4	+0.0
MgH_2_	−643.8	−15.0	+0.1
MgH_3_^−^	‐ 730.9	−13.5	+1.1
AlH_3_	−753.5	−10.9	+1.5
SiH_4_	−884.7	−10.4	+0.6
PH_3_	−930.8	−16.6	+0.0
SH^−^	−936.8	−24.0	+0.2
HCl	−952.9	−21.6	−0.7
FHF^−^	−1536.5	−30.6	+0.6
(H_2_O)_2_	−1508.3	−17.4	−0.1
(H_2_O)_3_	−2271.6	−24.4	−0.1

We now look into the reason for the abnormal behavior of BH_3_ and BeH_3_^−^. To try and understand whether this is compound‐specific behavior of BH_3_ or a property of boron in general we studied BH_4_^−^. A test where BH_4_^−^ was given the geometry and the basis set of CH_4_, resulted in the same small error reduction seen for BH_3_, whereas CH_4_ behaves normally. Thus, the behavior seems to stem from the nature of the basin around boron. In order to consider this behavior further, we increased the grid size. It turns out that the recovery error decreases significantly (from 0.44 to −0.02 kJmol^−1^) with the larger 15‐30 grid. This reduction (22‐fold) is typical and seen in all regular systems and it seems the anisotropic behavior of B is being picked up by the smaller grid.^40^ The situation of the iso‐electronic BeH_3_^−^ is more complicated than that of BH_3_. If the 15‐30 grid is employed then the 6D result is marginally more accurate than the 3D ESP result (+0.59 vs. + 0.60 kJmol^−1^). In fact one has to employ an angular grid larger than any used before in this work and coded “23” (770 angular grid points) before the 3D ESP approach gives an order of magnitude reduction in the recovery error over the 6D method. This great sensitivity to the angular grid size suggests that the element Be, like B, shows anisotropy in its basin when bonding.

Table 3 gives the CPU timings of the 6D and 3D ESP method, as well as the number of ESP integrals, for all systems given in Tables 1 and 2. The ratio of the timings between the two methods is only indicative (in principle) because (1) our computer cluster is heterogeneous in terms of its processors and (2) even if the jobs run on the same type of processor, that processor may have a different load due to other jobs running. Both these factors affect the timing. Table 3 shows that the ratio of the timings varies from 3 to 52, with most occurring in the range of 5–10 and with an average of very close to 10. However, these timings are for calculations employing one core, although the program is capable of being run in parallel and thus running much more quickly, but this will be reported in the future. The time taken to calculate the ESP integrals has not been taken into account. These calculations typically take from a few minutes to half an hour and involve the ESP integrals being written to disk. For example, for the water trimer there were just short of a billion ESP integrals to calculate (934,418,898) and this task was carried out in 92 groups (four groups running concurrently on the current batch system). All batches calculated the same number of integrals (10,264,674), other than the last one, which calculated 333,564 ESP integrals. The fastest group (excluding the last one), employing a single core, took 1 min 16 s of CPU time. However, the overall calculation can be even faster. Indeed, in calculating these ESP integrals, the G09 run includes the calculation of a Hartree–Fock wave function, prior to the computation of the required integrals. Technically, it is the ESP integrals over atomic basis functions that are needed. In other words, we do not require a wave function but only the atomic orbital basis and the grid positions. Thus, the wave function calculation is superfluous and clocks up unnecessary CPU time. Indeed, the wave function and 2PDM have already been generated in a previous calculation (prior to the ESP evaluation) and passed to MORFI. The resulting electron density, from this first run, is employed to generate a grid and it is these grid points that are passed back to G09 to have the atomic orbital ESP integrals evaluated at their location. Thus, the wave function is not needed for the ESP evaluation.

**Table 3:** The CPU timings (minutes) for each of the systems studied by the 6D and 3D approach, the ratio of the two timings rounded to the nearest integer and the number of ESP integral. All results are for the 10‐20 grid unless stated otherwise.

System	CPU Time: 6D MORFI	CPU Time: 3D ESP MORFI	CPU time ratio: 6D/3D	Number of ESP Integrals
LiH	118	3	39	16,951,998
BeH_3_^–^ a	963 (4599)	77 (89)	13 (52)	71,136,312 (220,443,132)
BH_3_ a	306 (2027)	28 (106)	11 (19)	71,238,888 (220,258,488)
CH_4_	766	148	5	117,963,345
NH_3_	358	26	14	71,538,648
H_2_O	92	10	9	38,663,685
HF	31	4	8	17,519,376
N_2_	101	14	7	41,050,836
O_2_	83	17	5	40,669,980
F_2_	109	17	6	40,248,318
Ne_2_	109	17	6	40,248,318
NO	83	17	5	40,756,716
CO	90	17	5	41,010,030
OF	109	14	8	40,533,960
NaH	108	28	4	37,881,570
MgH_2_	290	32	9	74,103,696
MgH_3_^−^	745	89	8	124,947,972
AlH_3_	822	316	3	124,947,972
SiH_4_	1358	197	7	193,862,505
PH_3_	1520	200	8	125,600,868
SH^−^	413	13	32	38,327,170
HCl	84	15	6	37,881,570
FHF^−^	323	39	8	77,000,922
(H_2_O)_2_	3971	328	12	283,381,584
(H_2_O)_3_ b	~24,000	3072	8	934,418,898

a The number that is not bracketed refers to the 10–20 grid while the bracketed number is for the 15‐30 grid, which is only employed for B and Be.

b The size of the water trimer meant that the full MORFI run had to be split into several runs. Variation in processor speed and other jobs running concurrently on the node affect the timings of each of these jobs. Thus, this timing is approximate and is a lower bound.

### Algorithm speed up (from 6D to 3D ESP) for a fixed recovery error

As mentioned in section Implementation of the 3D ESP algorithm, there are two ways of quantifying the progress made by the 3D ESP algorithm: (1) how the size of the grid affects the recovery error and (2) how much faster 3D ESP is compared to 6D for a fixed recovery error. Here, we look at the second way.

**Figure 2:**
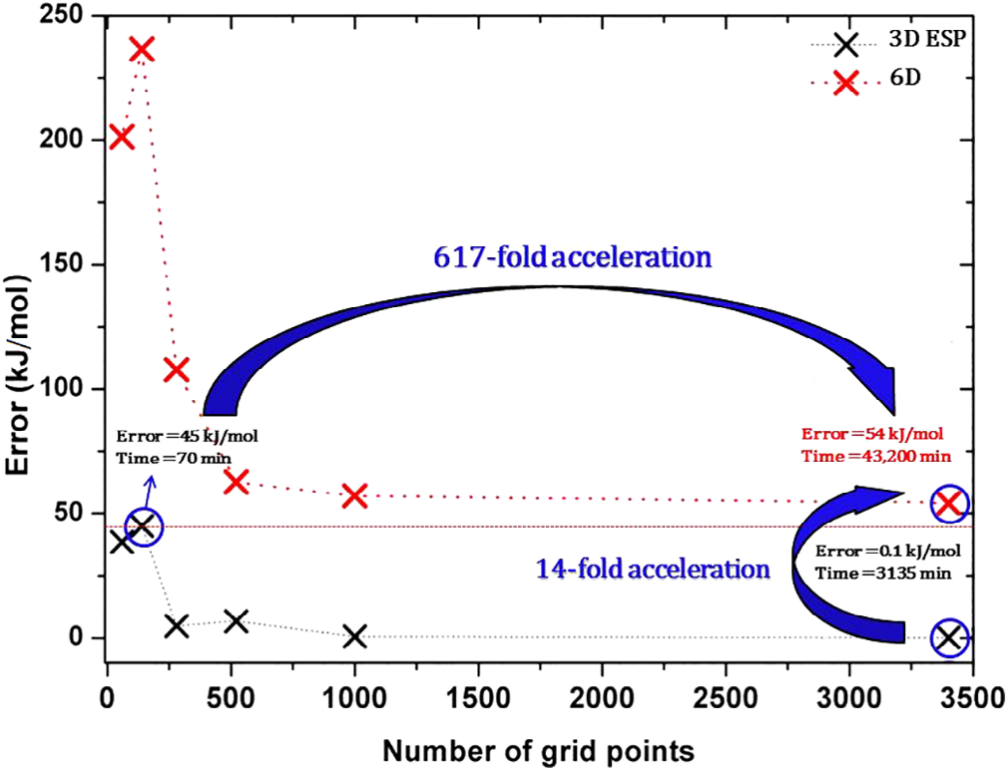
A comparison between the 6D and 3D ESP methods implemented in MORFI for the water trimer showing the recovery error (summed over all nine atoms), employing different grid sizes. The straight red dotted line marks the approximate recovery error at which the 6D and 3D ESP methods are compared. [Color figure can be viewed at http://wileyonlinelibrary.com]

The timings shown in Table 3 are impressive but still do not do justice to the improvements achieved by changing the algorithm from a 6D to a 3D ESP integration. Comparing the results using *the same grid* for both methods is fair and useful. Indeed, the very small recovery errors, often obtained by the 3D ESP method with small to medium grids, are impressive. However, for the purpose of constructing a training set for FFLUX, larger recovery errors are acceptable. Hence, much computational time can be saved by employing smaller grids by increasing the recovery error to a fixed value that we are happy with. Thus, we analyze the 3D ESP and 6D method by equalizing their respective recovery errors and then comparing the two CPU times. By doing this, it becomes possible to really appreciate the improvements achieved by the 3D ESP version of the code in terms of speed‐up. We carry out an analysis on a nontrivial system and one that FFLUX benefits from, given its ultimate aim to work for peptides in aqueous solution. Looking at the list of systems at hand, the choice then falls on the water trimer. In order to push the 3D ESP integration code to its absolute maximum, we aim for a recovery error of 0.1 kJmol^−1^ for the water trimer and search for the smallest grid available that can achieve this accuracy. However, as mentioned previously, realistically larger recovery errors will be tolerable in FFLUX, and the same procedure behind Figure 2 shall be carried out aiming for chemical accuracy, which is 4 kJmol^−1^.

Figure 2 contrasts the performance of the 6D and 3D ESP methods. Unfortunately the 6D method cannot reach a recovery error of ~50 kJmol^−1^ (see red dotted line in Fig. 2) with less than the maximum of 3500 grid points tested. Thus, this is the practical threshold (rather than 1 kJmol^−1^) allowing a direct comparison between the two methods. For the 6D method, it takes 43,200 min to reach this recovery error while for the 3D ESP method it takes 70 min, or 617 times less. These timings are obtained for two very different numbers of grid points: 6D needs many points while 3D ESP needs few. However, if the same grid size is employed by both methods then the speed‐up is about 14 (43,200 vs. 3135 min). This grid optimization also shows that while it is possible to achieve a recovery error as small as 0.1 kJmol^−1^ using ~3500 grid points, a still respectful error of 0.6 kJmol^−1^ can be achieved with only 1000 grid points, in about 150 min.

In principle, the grid size can be varied from atom to atom, as we have reported^27^ for water clusters by showing that hydrogen atoms required smaller grids in order to obtain good recovery errors compared to oxygen atoms. However, the implementation of the ESP 3D integration in MORFI had an unforeseen secondary effect: the required number of grid points to achieve small recovery errors is significantly lower when compared to the 6D integration. In fact, the ESP 3D approach makes the use of different grids for different atoms obsolete, because the required grids for both heavy and hydrogen atoms can be pushed to their lower limit.

**Figure 3:**
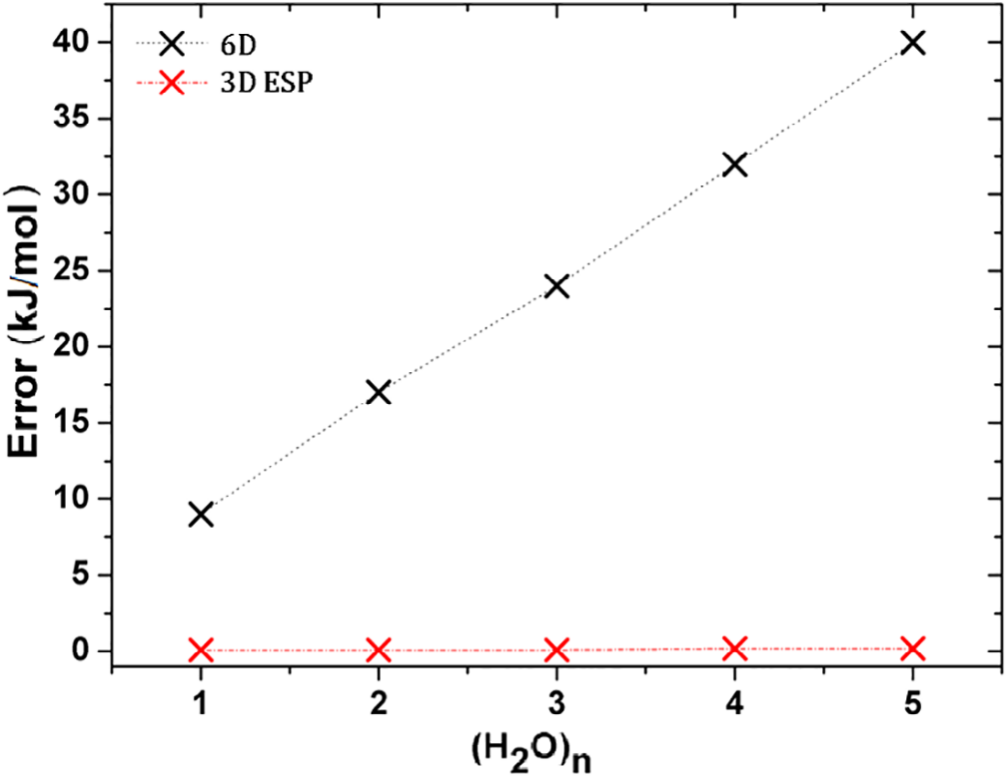
The recovery error for the 6D method increases linearly with the size of the system, while it remains relatively constant for the 3D ESP method. The grid used was 10–10 (3400 grid points) and gives recovery errors for the monomer, dimer, trimer, tetramer and pentamer of 0.1, 0.2, 0.1, 0.8, and 0.6 kJmol^−1^, respectively. [Color figure can be viewed at http://wileyonlinelibrary.com]

There is one final test to be done, which will further show the power of the 3D ESP method. We applied both the 6D and 3D ESP method to investigate a series of growing water clusters (H_2_O)_*n*_ (*n* = 1, 2,3, 4, and 5). Figure 3 plots the recovery error for each of these five water clusters. While the recovery error increases almost linearly with the number of atoms for the 6D method, this is not the case for the 3D ESP method. Once a sufficiently robust grid (10‐10, 3400 grid points) is used, the error remains more or less constant for the latter method. While the 6D recovery error starts off about 9 kJmol^−1^ for the water monomer, it becomes as large as 40 kJmol^−1^ for the water pentamer. However, for 3D ESP, the error remains around 0.1–0.2 kJmol^−1^ throughout the series, regardless of the system size. This result is superb for the incorporation of dynamic electron correlation (and thus dispersion) in FFLUX.

The 6D version of MORFI will remain relevant within the context of the quantum chemical topology literature, because it provides much physical insight, especially related to the nature of chemical bonds. Nonetheless, the fact that the recovery error obtained through the 6D algorithm heavily depends on the system size creates an unsustainable situation to transition FFLUX from small molecules to large biological systems. However, this is not the case for the 3D ESP algorithm because the recovery error is largely independent of the system size. One can use this size independency to the benefit of FFLUX, by first carrying out a grid optimization for, say, an amino acid and then apply the knowledge of this optimal grid to also obtain excellent recovery errors for polypeptides.

Regardless of how impressive the results look for the recovery errors, in chemistry, energy differences are usually the ultimate goal when it comes to predictions or calculations. These relative energies converge faster than the absolute energies themselves since the former benefit from error compensation. This effect cannot be easily seen in the results presented here (the errors are already very small), but it can be illustrated by the difference between the correlation energy of the water pentamer and that of five times the correlation energy of single water molecule, with the 6D “AB” integration code. The absolute recovery error for the pentamer is 7.7 kJmol^−1^ but the error is reduced to 5.5 kJmol^−1^ when considering energy differences.^27^

## Conclusions

The Møller–Plesset dynamic electron correlation of topological atoms can now be calculated 3–50 times faster for a given fixed size of quadrature grid. This is possible by replacing the numerical integration over the atomic volume by an analytical integration over whole space. The price paid is the loss of pairwise inter‐atomic electron energies. However, these are fortunately not required for the force field FFLUX, which only needs the correlation energy of a given atom interacting with all other atoms by summation. The total recovery error for all atoms in the water pentamer now amounts to only 0.6 kJmol^−1^ for a grid of only 3400 points. Moreover, for this system, the recovery error is largely independent of the system size.
